# Expanded Glass for Thermal and Acoustic Insulation from Recycled Post-Consumer Glass and Textile Industry Process Waste

**DOI:** 10.3390/ma16041721

**Published:** 2023-02-19

**Authors:** Luca Cozzarini, Lorenzo De Lorenzi, Nicolò Barago, Orfeo Sbaizero, Paolo Bevilacqua

**Affiliations:** 1 Department of Engineering and Architecture, University of Trieste, Via Valerio 6/1, 34127 Trieste, Italy; 2 Department of Mathematics and Geosciences, University of Trieste, Via Weiss 2, 34128 Trieste, Italy

**Keywords:** circular economy, expanded glass, glass recycling, acoustic insulation materials, thermal insulation materials

## Abstract

The production of glass foams obtained by recycling post-consumer glass and textile industry processing waste is presented. The mechanical, thermal and acoustic properties were characterized as a function of process temperature and time. The results showed that it is possible to produce glass foams with thermal and acoustic insulation properties from a mixture consisting of 96.5% of glass waste, 1% of textile waste and 2.5% of manganese dioxide, processed at temperatures between 800 and 900 °C for a time between 30 and 90 min. The samples had density in the range of 200–300 kg m^−3^, porosity of 87–92%, thermal conductivity of 85–105 mW m^−1^ K^−1^, noise-reducing factors of 0.15–0.40 and compressive strength of 1.2–3.0 MPa. Although their insulation performance was not as outstanding as that of polymer foams, these materials can emerge as competitive candidates for applications requiring non-flammability and high-temperature load bearing capacity in combination with low weight, mechanical strength, and thermal and acoustic insulation properties. The use of secondary raw materials (which accounted for 97.5% by weight of the synthetic blend) limits the energy required compared to that needed for the extraction, transportation and processing of primary raw materials, making these foams attractive also in terms of environmental footprint.

## 1. Introduction

The thermal comfort of enclosed spaces by heating, ventilation and air conditioning represents today 40% of energy consumption and 36% of carbon emissions in the European Union, making it one of the most energy-intensive commodity [1]. Similar values are also reported for the United States and the rest of the world [2,3]. Improved energy efficiency and proper building insulation are therefore critical to reduce the energy use, thus helping to reduce emissions associated with the combustion of fossil fuels [4,5]. Improving building thermal insulation would be also in line with the requirements of the European Commission’s energy performance of building directive [6]. Theoretically, the best thermal insulation is provided by vacuum; however, a more practical solution is the use of gases (such as air), which also show very low thermal conductivities (25–26 mW m^−1^ K^−1^) compared to liquids and solids [7]. To further increase the thermal insulation effect of gases, their volume can be divided into small cells, which cannot effectively transfer heat by natural convection. To achieve this, an artificial material can be shaped in a cellular foam of fibrous structures to trap and/or divide different gas volumes [8]. Nowadays, the insulation materials market is dominated by inorganic fibrous materials such as glass and mineral wool (GW, MW) and organic foamy materials such as expanded or extruded polystyrene (EPS) and foamed polyurethane (PU) [4,9,10]. These traditional insulators are produced from primary raw sources, such as minerals and fossil fuels. The use of secondary or renewable raw materials is now of critical concern to meet ecological and sustainability requirements, and the production of insulation materials from natural or recycled sources is being increasingly explored [11]. Some work has shown the possibility to recycle plastics or textile fibers into insulation panels, while others have focused on the use of bio-based or natural raw sources [12,13,14,15]. Other research has focused specifically on producing foams from recycled glass [16,17,18,19,20]. 

Glass is a very widespread material, with many uses, both in the industrial and in the domestic sectors. Its properties are modulated by its composition according to the intended use [21]. It is thus possible to obtain transparent or colored glass, or glass particularly resistant to thermal changes. Such wide use leads to the availability of large quantities of waste and recovery materials, also due to the increasingly widespread separate waste collection. These scraps often vary in size, color and contaminants. The different composition of glass makes it difficult to reuse its scraps for the production of new objects, since usually a very specific type of glass (with a controlled composition) is required [21,22]. Given the difficulty of reusing glass as a secondary raw material for the production of items with a high-quality standard, in order to reduce the disposal of glass in landfills, it is necessary to explore ways for its reuse for products for which neither a rigid compositional homogeneity of the starting material nor a beautiful final appearance is required. Finally, these products must have a market capable of absorbing their production, to give to the product an added value higher than the production costs. Within this framework, the production of glass foams and expanded glass from mixed glass waste may be an attractive option.

Glass foams are lightweight (density, 100–300 kg m^−3^) while retaining adequate mechanical strength; moreover, they are inert, chemically stable, not flammable and immune to biological degradation or animal feeding [23,24]. Their operating life is estimated from several decades to centuries. They are two-phase materials, composed of small gas-filled bubbles (gas phase), separated by very thin glass walls (solid phase). Glass foams can achieve a porosity as high as 90–95%, having a true potential both as heat-insulating and sound-absorbing porous materials. The intended uses for this material as an insulator layer, whose positioning is seldom visible, do not require good aesthetic features, and the mixing of different types of glass is merely a secondary problem. Thanks to these characteristics, expanded glass is being evaluated in many industrial sectors as a valuable solution for thermal and acoustic insulation, as they possess thermal, chemical and mechanical stability generally superior to that of polymer foams [24,25,26]. The main disadvantages of expanded glass are its higher unit price and higher thermal conductivity with respect to polystyrene foam or mineral fibers [25,27]. On the other hand, the use of secondary glass limits the energy required with respect to that necessary to extract, transport and process primary raw materials [26].

A promising solution is the production of glass foam in the form of expanded glass. Typically, a fine glass powder is first mixed with a foaming compound and other additives; the mixture is subsequently heated above the softening point of the glass, triggering the sintering of the glass particles and the release of gases from the foaming agent [28,29]. The gases expand in the softened glass, increasing the volume of the sample and leading to a porous, viscous mass. After cooling at room temperature, the porous structure is preserved, resulting in a lightweight, rigid material [27,30,31]. The foaming agent can release gases either by decomposition or by redox reactions. The most commonly used mineral foaming agents are carbonates, such as calcium, sodium and magnesium carbonate (CaCO_3_, Na_2_CO_3_ and MgCO_3_). These are thermally decomposed into calcium, sodium and magnesium oxides (CaO, Na_2_O and MgO), releasing carbon dioxide (CO_2_), which is responsible for glass foaming [29]. Since these oxides are already basic constituents of many silica glass formulations, the change of their ratios inside the glass can modify its characteristics (i.e., surface tension and/or viscosity). Carbon-based foaming agents, such as organic carbon compounds, can release CO_2_ upon oxidation while leaving no solid residues that could change the glass properties [23,27]. Some studies focused on the use of alternative and natural sources as foaming agents, such as eggshells [23,32], oysters [33] plant parts and leaves [34,35]. Some authors showed the possibility to use calcium sulfate from the ceramic industry waste [36], silicon carbide [37,38,39] and silicon nitride [40]. In this research work, we show and discuss the production of foamed glass starting from a mixture composed of 97.5% of secondary raw materials (96.5% post-consumer glass waste and 1% process waste from the synthetic textile industry) and only 2.5% of primary raw materials (manganese IV oxide, MnO_2_). The mechanical, thermal and acoustic properties were characterized as a function of production parameters (temperature and time). Differently from other works, the commingled glass originated from municipal waste collection rather than from a specific type of glass (i.e., windows, panels, CRT screens, etc.) and was not sorted by type, composition or color. Synthetic textile process waste was used as the carbon-based pore-forming agent, while MnO_2_ was used as the oxidant. The use of secondary raw materials avoids the landfilling of glass and allows also the recycling of industrial waste as a valuable and inexpensive carbon source. Thanks to its good properties and its fabrication process based on a circular economy approach, this material could be appealing for thermal and acoustic insulating applications in building and industrial applications also in terms of environmental footprint [41].

## 2. Materials and Methods

### 2.1. Foam Production

Manganese IV oxide (MnO_2_, reagent-grade, micrometric size) from Carlo Erba Reagents was used as purchased. Mixed glass from post-consumer items (bottles, food containers, etc.) was obtained from municipal glass waste sorting. The glass was washed and then reduced to a granulate by jaw crushers and cylindrical mills (SAIMA – Speciali Apparecchiature Industriali Meccaniche e Affini, Milano, Italy), obtaining a material with particle size between 1.5 and 2.8 mm by sieving. Glass density was determined with a Gay-Lussac pycnometer.

The chemical analysis of the glass was performed by a portable X-Ray Fluorescence (pXRF) instrument (Olympus Vanta C Series, Evident Scientific, Waltham, MA, USA) with a 4 W Ag anode X-ray tube, a silicon drift detector (SDD) and an excitation source ranging from 8 to 50 keV.

The industrial process waste (in the form of sludge) was obtained from a synthetic textile production plant. This process waste was dried at 110–120 °C for 24 h and pulverized to obtain a fine powder. This powder was characterized by means of Fourier transform infrared spectroscopy (FT-IR) with a Thermo-Nicolet Nexus 470 spectrometer, equipped with a diamond crystal attenuated total reflection (ATR) accessory. The spectra were acquired at a resolution of 2 cm^−1^ over a spectral range from 600 to 4000 cm^−1^.

The foaming mixture was prepared by blending 96.5% wt. of glass granulate, 1% wt. of dried textile waste powder and 2.5% wt. of MnO_2_. This mixture was pulverized with a Herzog mill for 70 s, obtaining a fine powder (D_50_ and D_90_ particle sizes in the range of 18–20 μm and 85–95 μm, respectively). The granulometric curves were determined by light scattering techniques with a Malvern Mastersizer 2000E analyzer. The mixture (125 g) was inserted in a square steel mold (80 × 80 mm^2^); it was first leveled manually (slight compression with the top of the mold) and then pressed by applying a 255 kN load for three minutes with a Weber PW40 hydraulic press. A green of 80 × 80 × 11 mm^3^ was obtained. The green was heated (10 K min^−1^) in a FALC FM13 muffle, at different temperatures (800, 850, 875 and 900 °C) and for different times (30, 45 and 90 min). After the scheduled sintering time, the samples were quickly extracted from the furnace for rapid cooling (down to 550 °C–600 °C) and then were cooled slowly in hot air for 2–3 h down to room temperature inside the furnace, which was meanwhile turned off. Rapid cooling was necessary to freeze the expanded structure, causing the viscosity of the glass to increase suddenly, while the subsequent slow cooling prevented the breakage of the specimens due to thermal contraction stresses. The samples were named after the process temperature and time (i.e.,: sample “800_30”: sintered at 800 °C for 30 min; sample “850_45”: 850 °C for 45 min, and so on).

### 2.2. Samples Dimension, Mass and Density

The sintered samples were cut into regular geometrical shapes (square-based or cylindrical). The dimensions were measured with a digital caliper (RS pro, code 841-25), rounding the value to 10^−1^ mm (average of three measurements for each dimension). The mass was determined with a digital balance (Sartorius CP244S), rounding the value to 10^−1^ g, while the volume was calculated as the product of the three dimensions (in the case of the square-based samples) or according to πR^2^H (with R being the radius, and H the height) for the cylindrical samples. The density of the samples (rounded value to kg m^−3^) was calculated by dividing their mass by their volume.

### 2.3. Porosity Characterization

A volume of 15 × 15 × 15 mm^3^ was extracted from the center of each representative sample and characterized by X-ray microcomputed tomography (μCT). The acquisitions were performed by means of a custom-made cone-beam system (TOMOLAB, Elettra-Sincrotrone Trieste), with resolution of 8 μm, beam energy of 40 kV and intensity of 200 μA, with an exposure time of 2.5 s. Three-dimensional slices were reconstructed and processed with FIJI package of ImageJ2 software.

### 2.4. Mechanical Tests

The compression tests were performed using a Shimadzu AGS-X 10 dynamometer (10 kN load cell). The test speed was set to 1.5 mm min^−1^, while the signal acquisition time was set to 0.25 s. The mechanical properties (compression modulus *E* and maximum strength *σ_M_*) were determined according to ASTM C165, procedure “A” [42]. The compression toughness was determined as the area under the stress–strain curve. Each data point represents the average of five specimens.

### 2.5. Sound Absorption Properties

A two-microphone plane wave impedance tube (Kundt’s tube) was used to determine the sound absorption properties of the samples, according to the ISO 10534-2 standard [43]. Three cylindrical samples (diameter 44 mm; thickness 18 mm) were tested for each sintering condition.

### 2.6. Thermal Conductivity

Thermal conductivity was measured with a Netzsch HFM 446 heat flow meter on the square-based samples (100 × 100 × 18 mm^3^) according to the technical standard ASTM C518 [44], at an average temperature of 25 °C. Three samples were tested for each sintering condition.

## 3. Results and Discussion

### 3.1. Characterization of the Raw Materials

The measured density of the glass powder was 2510 ± 7 kg m^−3^. The chemical analysis of the glass powder is reported in Table S1 of Supplementary Material; the grain size distribution curves and some percentiles of the mixture powder are reported in Figure S1 and Table S2 of Supplementary Materials. The FT-IR spectrum of industrial textile waste is reported in Figure S2 of Supplementary Material. The industrial textile waste was identified as a terephthalic acid salt [45], a typical monomer in the production of aromatic polyesters such as polyethylene terephthalate (PET), frequently utilized in the synthetic textile industry.

### 3.2. Sample Production

The combination of a carbon-based foaming agent and an oxidant reagent has already proven to be an effective way to produce low-density foamed glass [27,46]. In our work, we used organic molecules derived from industrial synthetic textile production waste, with the addition of MnO_2_. Organic molecules oxidize quickly in the presence of air at high temperatures (800–900 °C); nevertheless, these carbon-containing compounds were exposed to air only on the surface of the foaming mixture. The organic molecules inside the mixture were insulated from the air by the soften glass, and, since carbon oxidation requires a suitable amount of oxygen, they most likely underwent pyrolysis and carbonization. It was already shown that manganese IV oxide (MnO_2_) acts both as a foaming agent and as an oxidant, since it thermally decomposes at lower oxidation states releasing oxygen gas, which can contribute to the foaming process and also oxidize carbon molecules [27]. The decomposition of MnO_2_ happens in two steps: first, around 600 °C, it reduces to manganese III oxide (Mn_2_O_3_); then, around 900–950 °C, it further reduces to manganese II oxide (MnO) [47]. The carbon molecules react with oxygen, thus releasing CO_2_ gas, which acts as a foaming agent. Due to the gas production, pores emerge inside the softened glass mixture, providing a porous structure.

### 3.3. Sample Properties

**Figure 1:**
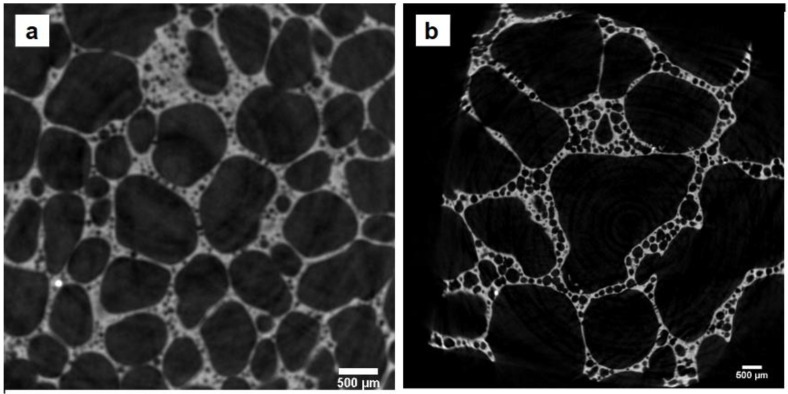
Representative slices of the micro-CT scans of two samples: 800_30 (**a**) and 900_90 (**b**).

**Figure 2:**
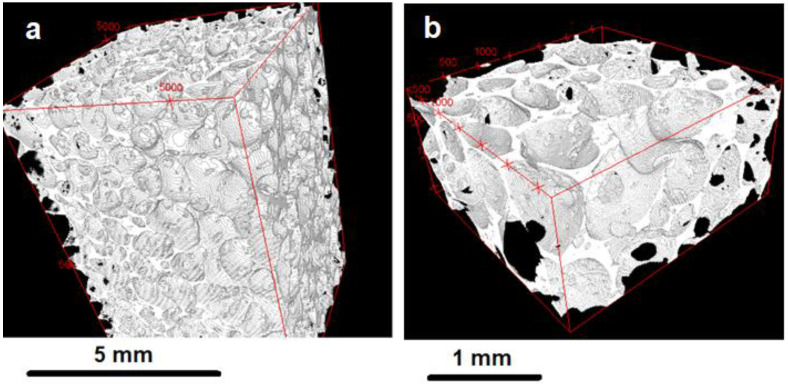
Representative 3D volumes reconstructed from the micro-CT data: sample 800_30 (**a**) and sample 900_90 (**b**).

Upon visual inspection, the overall structure of the material appeared as a foam of glass, with thin walls separating macroscopic pores. Representative pictures of the samples and their porosity are reported in Figures S3 and S4 in Supplementary Materials. Representative slices of samples from the µ-CT scans are shown in Figure 1; Figure 2 shows two representative 3D-reconstructed volumes from the µ-CT analysis (additional macrographs, micrographs and 3D volume models of all the samples are available on request). It can be seen that, additionally to the primary macro porosity, smaller secondary pores were found within the cell walls. 

The samples’ density, thermal conductivity, mechanical and acoustic properties are reported in Table 1.

### 3.4. Density

**Figure 3:**
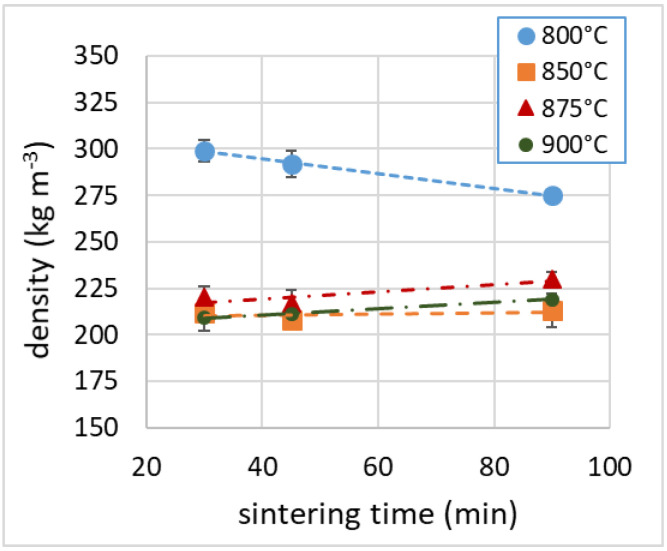
Sample density as a function of the sintering time.

The density of the samples sintered between 850 and 900 °C lay in the range of 210–230 kg m^−3^, while the samples produced at 800 °C had higher densities (275–300 kg m^−3^). For comparison, rock and glass wools are lighter (100–150 kg m^−3^) than our expanded glass samples, and polymeric foams are even much lighter (15–20 kg m^−3^). Considering an average density of soda–lime glass of 2500 kg m^−3^ (2400–2800 kg m^−3^) [48], which is in line with the measured density of the glass powder used in this work (2510 ± 7 kg m^−3^), we assumed a porosity (as volumetric void percentage) between 88% and 91%. Similar results of porosity values were determined by µ-CT, as reported in Table 2. The density values (as seen in Figure 3) appeared to be slightly correlated with the process time, with opposite trends: the density decreased with the sintering time for samples sintered at 800 °C, while it increased modestly with the sintering time for the other samples.

### 3.5. Porosity 

**Figure 4:**
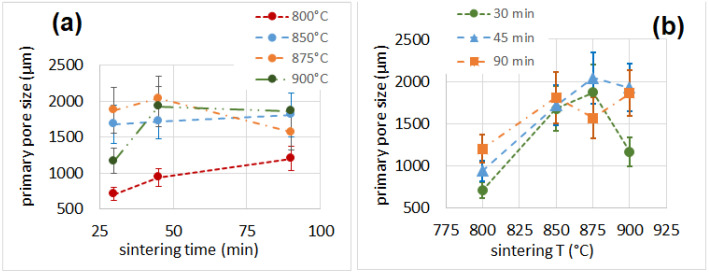
Primary pore size as a function of sintering time (**a**) and temperature (**b**).

**Figure 5:**
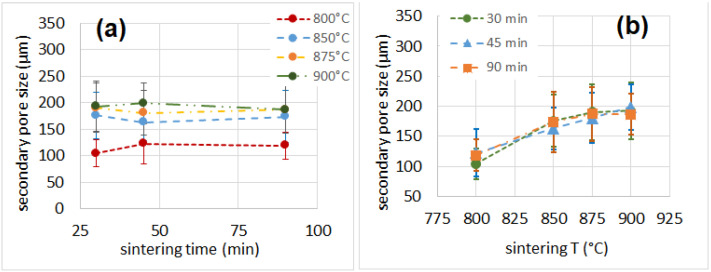
Secondary pore size as a function of sintering time (**a**) and temperature (**b**).

The primary and secondary pore sizes are reported in Table 2 and Figure 4 and Figure 5. The thickness of the cell walls is reported in Table 2. The percentage of porosity, determined as the complementary of the solid volume fraction calculated after µ-CT analysis, is also reported in Table 2. The size of the primary pores seemed to increase with the sintering time for samples produced at 800 °C and to slightly increase with the sintering time for samples produced at 850 °C. Conversely, the size of the primary pores for samples produced at 875 °C and 900 °C showed the maximum value at a processing time of 45 min. On the other hand, the size of the secondary pores did not seem to be influenced by the sintering time, but increased with the process temperature. The porosity lay in the same range (85–89%) for all samples except for those produced at 800 °C for 30 min, for which the value was lower (81%). Porosity can be closed (i.e., the cells are isolated from each other) or open. In the first scenario (porosity predominantly closed), the material shows good thermal insulation properties; in the second scenario (porosity predominantly open), the thermal insulation properties are slightly reduced, but the sound insulation capabilities are improved [30,49]. 

The production of foamed glass is controlled by many factors (glass composition, particle size, additives, process temperature, etc.). Once the characteristics of the glass and additives are settled, the process temperature is the key parameter that influences the foam properties, since the density and type of porosity are directly determined by the glass viscosity, the rate of bubble formation and coalescence [25,28,50]. Glass viscosity η is a function of the temperature and of the parameters *A*, *B* and *T_0_* [48]:

(1) $$log\;\left(\eta \right)=A+\frac{B}{T-T_{0}}$$

For soda–lime glass, we assume A = −2.464, B = 3828 and T_0_ = 272.7 °C [48]. Therefore, η = 6 × 10^4^ Pa∙s (800 °C), 1.4 × 10^4^ Pa∙s (850 °C), 7.5 × 10^3^ Pa∙s (875 °C) and 4.2 × 10^3^ Pa∙s (900 °C). It can be noticed that the glass viscosity values at 850, 875 and 900 °C are about 24%, 13% and 7% of the viscosity value at 800 °C. It is also known that higher temperatures (which provide lower viscosities) lead to the rapid formation and coalescence of gas bubbles, resulting in foam collapse. From these premises, it can be assumed that the higher glass viscosity of the sample produced at 800 °C led to smaller pores and a closed-cell structure.

### 3.6. Mechanical Properties

**Figure 6:**
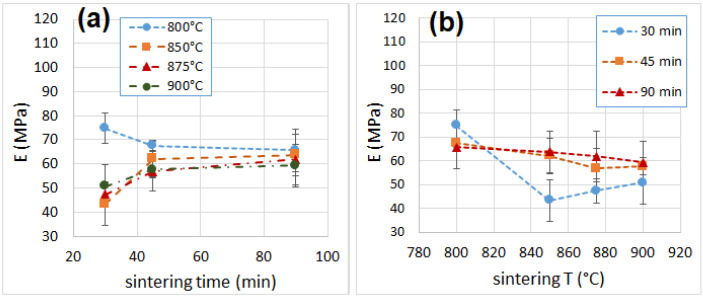
Compressive modulus as a function of sintering time (**a**) and temperature (**b**).

**Figure 7:**
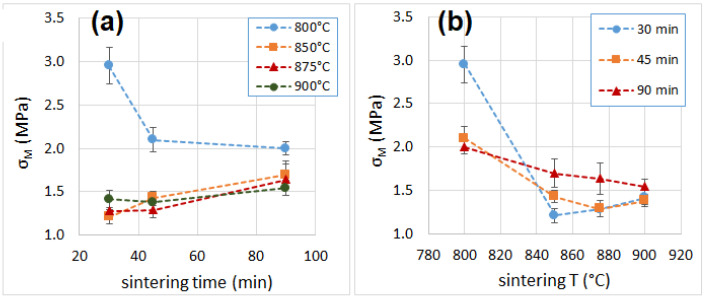
Compressive strength as a function of sintering time (**a**) and temperature (**b**).

The average curves of the compression tests are shown in Figure S5 of Supplementary Material. The values of modulus, strength and toughness determined after the compression tests are reported in Table 1. All samples showed mechanical properties that were superior to those of polymer foams (EPS and PU) [51,52,53,54] and outperformed by far those of mineral-based insulation materials (glass wool and mineral wool) [55], which will make them particularly suitable for load-bearing applications. The compressive modulus (Figure 6 and Figure 7) increased from 30 to 45 min of sintering time, while it remained unchanged after increasing the sintering time from 45 to 90 min. This behavior was observed for samples processed at 850 °C or higher, while the trend was the opposite for the sample at 800 °C, which showed a reduction of the compressive modulus from 30 to 45 min of sintering. The modulus then remained constant when increasing the sintering time from 45 to 90 min. This behavior can be explained by the very small pore size of the sample sintered for 30 min, when compared with the pore size of other samples at longer sintering times. This condition negatively affects the density, but enhances the mechanical strength.

**Figure 8:**
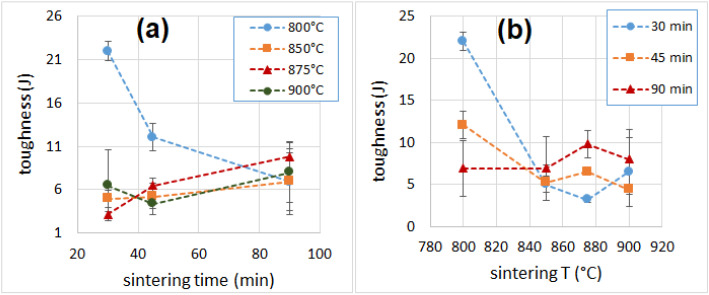
Compressive toughness as a function of sintering time (**a**) and temperature (**b**).

**Figure 9:**
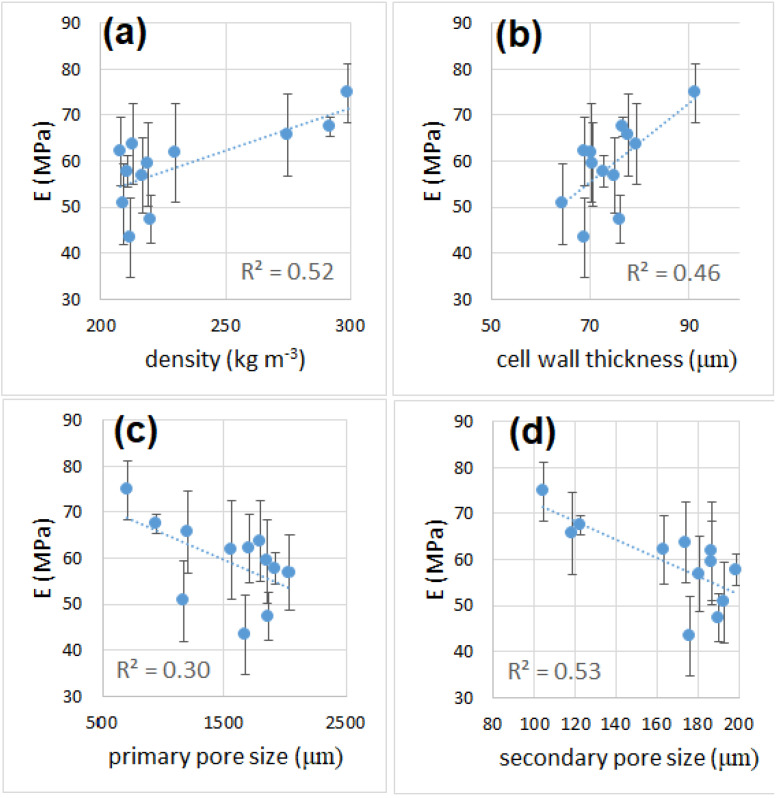
Compressive modulus as a function of density (**a**), cell wall thickness (**b**), primary pore size (**c**) and secondary pore size (**d**).

**Figure 10:**
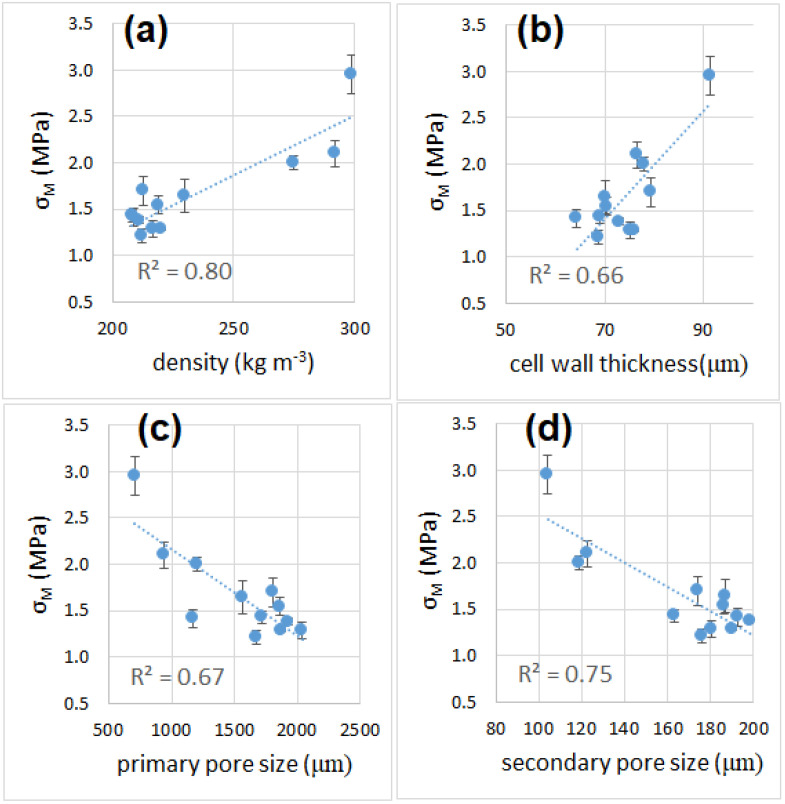
Compressive strength as a function of density (**a**), cell wall thickness (**b**), primary pore size (**b**) and secondary pore size (**b**).

The best mechanical performances, in terms of strength and modulus, were recorded for the samples produced at 800 °C for 30 min. The specimens’ breaking behavior was highly variable (some sample failed under a 10–15% strain, others resisted up to a 55% strain), but none of them was truly brittle (in fact, no specimen failed suddenly, as can be noticed from the curve reported in Supplementary Materials, Figure S5.). The overall toughness is indicated by the successive failure of individual cell structures (whose walls, made of glass, are brittle). The absorbed compressive energy (Figure 8), which can be directly related to the toughness of a sample, was higher for specimens produced at 800 °C and shorter times (22 J and 12 J, for samples sintered at 800 °C for 30 min and 45 min, respectively), while all the other samples showed an average absorbed energy between 3 and 10 J. Figure 9 and Figure 10 reports values of compressive modulus and strength as a function of density, cell wall thickness and pore size.

The values of E, as expected, seemed to be loosely related to the density: heavier samples were stiffer, as seen in Figure 9a. However, the relation is more complex, since the type of cell structure (open or closed porosity) and the presence of secondary porosity can also influence the modulus. From the Gibson–Ashby model [56,57,58], the elastic modulus of the cellular material can be related to the density and the type of porosity:

(2) $$\frac{E}{E_{o}}={\left(\frac{\rho }{\rho_{0}}\right)}^{2}\frac{1}{{\left(1+\varphi \right)}^{2}}\left[1+\frac{\rho }{\rho_{0}}\frac{\varphi^{3}}{1+\varphi }\right]$$

where *E* is the elastic modulus of the cellular material, *E_0_* is the elastic modulus of the bulk material (for the average soda–lime glass, 70 GPa [48]), ρ is the density of the foam, and *ρ_0_* is the density of the glass (2500 kg m^−3^ [48]). *φ* is a parameter defined as the ratio of the volume of material on the faces to that on the edges. For an open-cell material, *φ* = 0; for a closed-cell material, *φ = l/t*, where *l* is the length of the array members of the cell, and *t* is their thickness. In practical terms, the material behaves as an open-cell foam when *φ* < 1, and as a closed-cell foam when *φ* > 5. From the experimentally determined moduli, it was possible to derive the value of *φ* for each sample, inferring the type of porosity (open or close), as reported in Table 3, where E_1_* (*φ* = 1) is the theoretical value of E, calculated with Equation (1), assuming a value of *φ* = 1 (nearly open-cell structure). E_5_* (*φ* = 5) is the theoretical value of E, calculated with Equation (1), assuming a value of *φ* = 5 (closed-cell structure).

φ* is the value of *φ* to be input in Equation (1) to obtain a theoretical value of E equal to that obtained experimentally from the compression tests. A value of *φ* * close to 1 is an indication of an open-cell structure, while a value of *φ* * close to (or greater than) 5 suggests a closed-cell structure. It is possible to notice that the samples obtained at lower temperatures (particularly those sintered at 800 °C) showed higher *φ* * values, while those obtained at higher temperatures had lower φ* values. Moreover, longer process times seemed to result in lower φ* values (favoring an open-cell structure). As discussed earlier, the mechanical properties were quite similar for samples produced at 850, 875 and 900 °C, while they were superior for samples produced at 800 °C (with the best performances recorded for samples produced at 800 °C for 30 min). This can be explained by their higher density and the prevalence of a closed-cell structure, deduced from the φ* values calculated above (Table 3).

### 3.7. Thermal Insulation Properties

**Figure 11:**
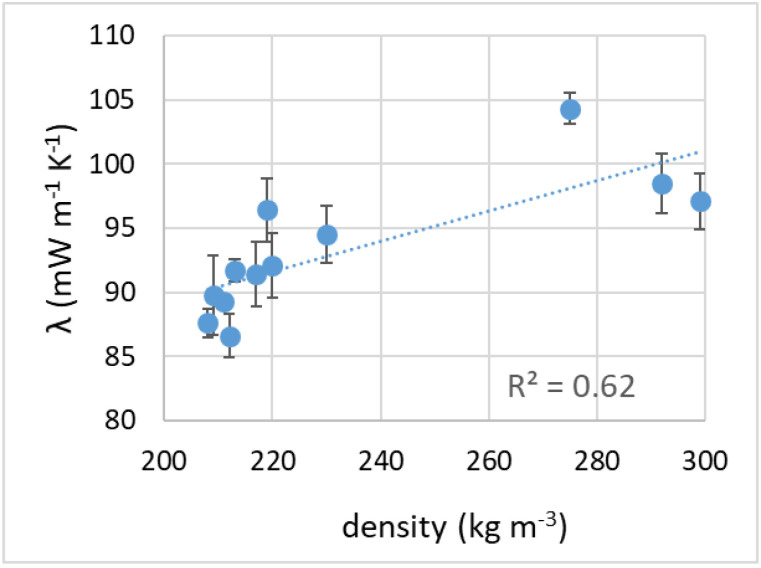
Thermal conductivity as a function of density.

**Figure 12:**
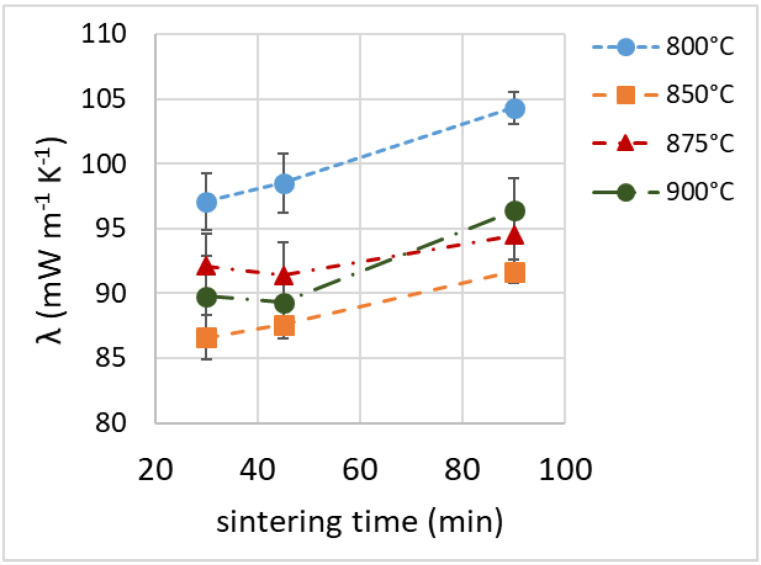
Thermal conductivity as a function of sintering time.

The thermal conductivity (λ, reported in Table 1) ranged from 86 to 104 mW m^−1^ K^−1^. It appeared to be only loosely related to the density of the samples (with heavier samples showing higher conductivity, as seen in Figure 11). At all process temperatures, the thermal conductivity values seemed to increase moderately with the sintering time (the samples foamed for 90 min showed the highest conductivity, as seen in Figure 12).

For comparison, rock wool, glass wool, expanded polystyrene or polyurethane foam perform better: their values of thermal conductivity, ranging from 20 to 30 mW m^−1^ K^−1^ [4,51,52,59], are about from 1/3 to 1/5 of those registered for our expanded glasses. The thermal conductivity should be affected by density and porosity type, with a closed-cell structure favoring thermal insulation. Thermal conduction in the solid phase, related to the density of a sample, contributes to most of the overall thermal conductivity, while conduction in the gas phase is greater in an open-cell structure, due to the different gas composition (air instead of CO_2_) and to the possible convective heat transfer [7,49].

### 3.8. Acoustic Properties

**Figure 13:**
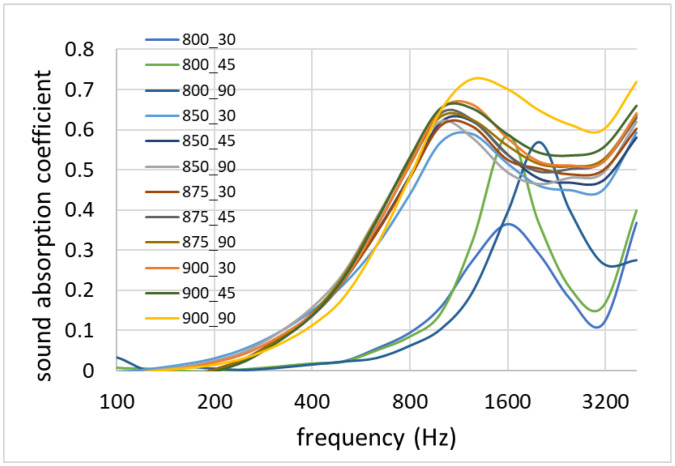
Sound absorption coefficient as a function of frequency for the different samples.

The average sound absorption coefficient curves as a function of sound frequency are reported in Figure 13; the noise reduction coefficient (NRC) values are reported in Table 1. It can be noticed that all samples has a low sound absorption at low frequencies. As the sound frequency increased, the absorption coefficient rose until it reached a value of 0.5 above 800 Hz. As the frequency increase further, the coefficient remained high, particularly in the range of the frequencies best heard by the human ear, indicated between 2000 and 5000 Hz. For the samples sintered at 800 °C, the absorption properties were worse compared to the other samples, and the absorption maximum was located at higher frequencies (1600–2000 Hz). The samples produced at 850–900 °C appeared to have a very similar acoustic performance (apparently unrelated to processing temperature or time), while the worst performing samples (with completely different curves and behavior) seemed to be those produced at 800 °C. The acoustic performance can be related, again, to both density and porosity type (with open porosity providing better acoustic insulation performances). The closed-cell structure of the samples sintered at 800 °C was likely responsible for their poor acoustic performance.

### 3.9. Process Parameters

The thermal and acoustic properties were significantly better for samples produced at 850, 875 and 900 °C with respect to samples produced at 800 °C. However, since there were no substantial differences between the samples produced at 850 °C for intermediate times (45 min) and those produced at 900 °C for long times (90 min), from an energy-saving and optimization perspective it would be meaningless to spend energy and time (900 °C for 90 min), as the samples produced at 850 °C for 45 min already achieved satisfactory performances.

Aiming at optimizing the thermal and acoustic properties, the samples sintered at 850, 875 and 900 °C for different firing times were comparable. This implies that the production at 850 °C for 45 min was the most cost-effective one. On the other hand, if the mechanical property of compressive strength is considered, the best result was provided by the samples sintered at 800 °C for 30 min. In general, for the purpose of economic feasibility of the process, it is better to favor shorter times (higher production) than lower temperatures (energy saving).

## 4. Conclusions

The results showed that it is possible to produce glass foams from a mixture composed of 96.5% *w*/*w* of blended glass waste, 1% *w*/*w* of textile waste and 2.5% of MnO_2_ *w*/*w*, sintered at a temperature between 800 and 900 °C for a time between 30 and 90 min. The products had a density between 200 and 300 kg m^−3^, a porosity between 81% and 90%, a thermal conductivity between 85 and 105 mW m^−1^ K^−1^, noise-reducing factors between 0.15 and 0.4 and a compressive strength between 1.2 and 3 MPa. These materials are attractive candidates for applications that require a good combination of low weight, thermal/acoustic insulation and mechanical strength. Although the insulation performance was not outstanding when compared with that of other types of insulation materials (such as polymeric foams), in load-bearing applications where stiffness, mechanical strength, chemical inertia, non-flammability and high-temperature resistance are crucial, the glass foams investigated in this study may emerge as competitive alternatives. Moreover, in the next future, a manufacturing process able to reduce energy consumption, raw material use and CO_2_ emissions will be strategic and will assume a significant value. The production method used for the samples examined in this study, starting from secondary raw materials, allows reducing the energy required compared to that needed for the extraction, transportation and processing of raw materials, with obvious benefits in terms of environmental indexes.

## Figures and Tables

**Table 1:** Samples’ properties. λ: thermal conductivity; NRC: noise reduction factor; E: compression modulus; σ_M_: compression strength.

Sample	Density (kg m^−3^)	λ (mW m^−1^ K^−1^)	NRC	E (MPa)	σ_M_ (MPa)	Toughness (J)
800_30	299 ± 6	97.1 ± 2.2	0.15	74.8 ± 6.4	2.95 ± 0.21	22.0 ± 1.1
850_30	212 ± 3	86.6 ± 1.7	0.35	43.3 ± 8.7	1.21 ± 0.08	5.0 ± 1.0
875_30	220 ± 6	92.1 ± 2.5	0.35	47.3 ± 5.2	1.28 ± 0.01	3.2 ± 0.3
900_30	209 ± 7	89.8 ± 3.1	0.35	50.7 ± 8.8	1.42 ± 0.10	6.5 ± 4.1
800_45	292 ± 7	98.5 ± 2.3	0.15	67.5 ± 2.1	2.10 ± 0.14	12.1 ± 1.6
850_45	208 ± 4	87.6 ± 1.1	0.35	62.0 ± 7.4	1.43 ± 0.07	5.2 ± 2.0
875_45	217 ± 7	91.4 ± 2.5	0.35	56.8 ± 8.2	1.29 ± 0.09	6.5 ± 0.3
900_45	211 ± 4	89.3 ± 0.4	0.35	57.7 ± 3.5	1.38 ± 0.04	4.4 ± 0.6
800_90	275 ± 2	104.3 ± 1.2	0.15	65.6 ± 8.9	2.00 ± 0.08	6.9 ± 3.3
850_90	213 ± 9	91.7 ± 0.9	0.35	63.7 ± 8.8	1.70 ± 0.16	7.0 ± 3.8
875_90	230 ± 4	94.5 ± 2.2	0.35	81.8 ± 10.7	1.64 ± 0.18	9.8 ± 1.6
900_90	219 ± 1	96.4 ± 2.5	0.40	59.3 ± 9.0	1.55 ± 0.09	8.0 ± 3.5

**Table 2:** Mean pore size and % of porosity determined by micro-CT.

Sample	Cell Wall Thickness (µm)	Primary Pore Size (µm)	Secondary Pore Size (µm)	Porosity %
800_30	91 ± 14	707 ± 95	104 ± 26	81.1
850_30	69 ± 10	1677 ± 268	176 ± 44	88.4
875_30	76 ± 11	1873 ± 322	190 ± 46	88.6
900_30	64 ± 9	1169 ± 322	193 ± 47	86.8
800_45	77 ± 11	941 ± 124	123 ± 39	83.7
850_45	69 ± 10	1716 ± 239	163 ± 35	88.8
875_45	75 ± 11	2040 ± 308	181 ± 42	89.2
900_45	73 ± 11	1925 ± 283	200 ± 38	89.9
800_90	79 ± 11	1202 ± 165	119 ± 26	85.3
850_90	79 ± 18	1808 ± 302	174 ± 50	87.5
875_90	70 ± 10	1569 ± 249	187 ± 44	87.7
900_90	71 ± 10	1860 ± 269	187 ± 34	89.5

**Table 3:** Density, experimental and theoretical values of the compressive modulus (Gibson-Ashby model). E*^1^ and E*^5^ are the values of the modulus calculated for *φ* = 1 and *φ* = 5.

Sample	Density (kg m^−3^)	E (MPa)	E*^1^ (*φ* = 1) (MPa)	E*^5^ (*φ* = 5) (MPa)	Φ*
800_30	299	74.8	285	107	>5
850_30	212	43.3	121	32	3.8
875_30	220	47.3	131	38	3.8
900_30	209	50.7	118	34	2.8
800_45	292	67.5	262	96	>5
850_45	208	62.0	116	33	2.2
875_45	217	56.8	127	37	2.7
900_45	211	57.7	120	34	2.4
800_90	275	65.6	225	70	>5
850_90	213	63.7	122	35	2.2
875_90	230	61.8	143	43	2.9
900_90	219	59.3	129	38	2.6

## Data Availability

The data presented in this study are available within the article and in Supplementary Material. Further data are available on request from the corresponding author.

## Supplementary Materials

The following supporting information can be downloaded at: https://www.mdpi.com/article/10.3390/ma16041721/s1, Table S1: chemical analysis of post-consumer glass powder (secondary raw materials) by XRF; Table S2: characteristic diameters of the synthesis mixture powder from the granulometric analysis; Table S3: vibrational bands of the FTIR spectrum of textile industrial scraps; Figure S1: granulometric curve of the synthesis mixture powder; Figure S2: FTIR spectrum of textile industrial scraps; Figure S3: macrographs of the different samples; Figure S4: representative pictures of some of the produced samples, before and after cutting into the final size; Figure S5: compression stress/strain curves of different samples.
