# Supplementary material for: Expanded Glass for Thermal and Acoustic Insulation from Recycled Post-Consumer Glass and Textile Industry Process Waste
Source: Materials (Basel). 2023 Feb 19;16(4):1721. doi: 10.3390/ma16041721 (PMC9961893; doi:10.3390/ma16041721)
Supplement: Supplementary file 1 [file materials-16-01721-s001.zip › materials-2181804-supplementary.pdf]

Table S1 chemical analysis of post-consumer glass powder (secondary raw materials) by XRF

oxide	wt %
SiO_2	74.14 ± 0.50
TiO_2	0.05 ± 0.01
Al_2O_3	0.57 ± 0.31
Fe_2O_3	0.22 ± 0.11
MnO	0.02 ± 0.01
CaO	9.78 ± 0.31
K_2O	0.66 ± 0.05
P_2O_5	0.11 ± 0.01
BaO	0.06 ± 0.01
Cr_2O_3	0.04 ± 0.03
PbO	0.01 ± 0.01
others*	14.32 ± 0.54

* "Others" represents the sum of light elements. After revieving several literature sources, this value can be related mostly to the sum of sodium and magnesium oxides (Na_2O + MgO)

Table S2 characteristic diameters of synthesis mixture powder from granulometric analysis

Granulometric value	Size (μm)
D10	3.5
D16	4.9
D25	7.3
D50	18.8
D75	48.0
D84	69.0
D90	90.9

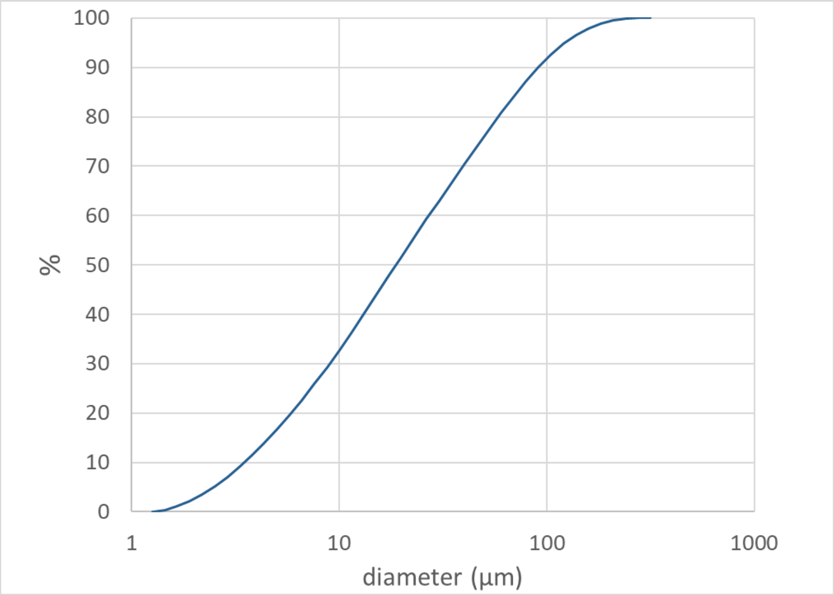

Figure S1 granulometric curve of synthesis mixture powder

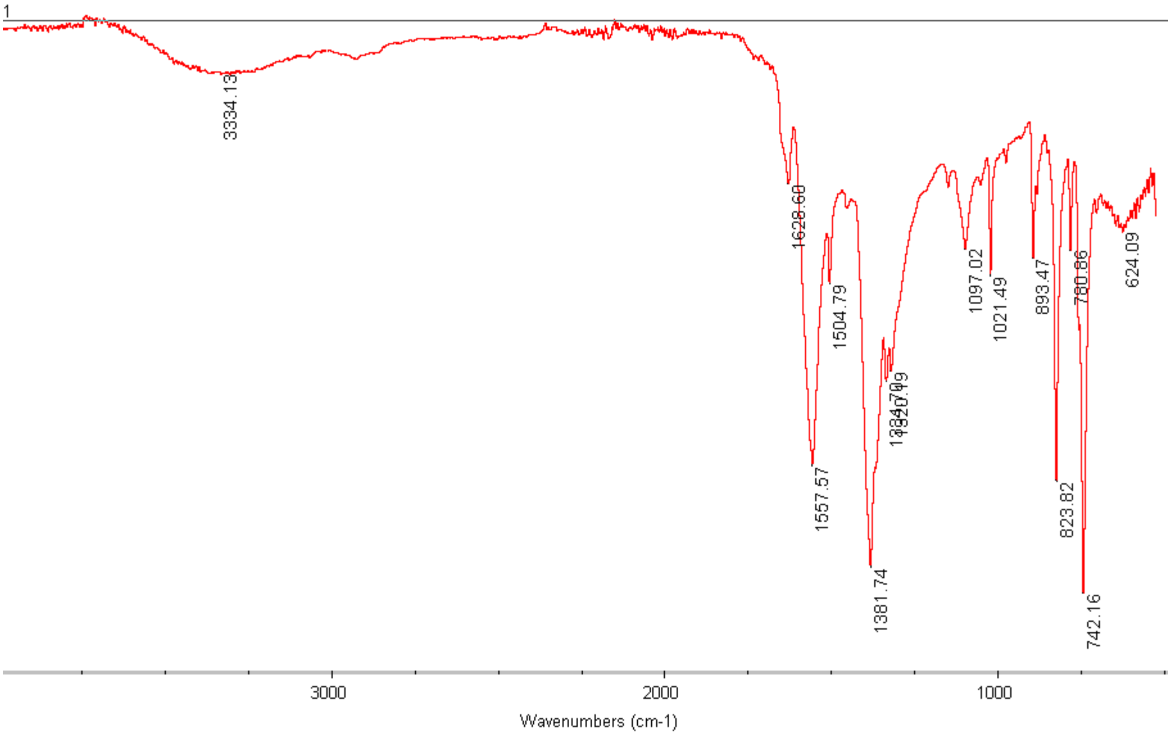

Figure S2 FTIR spectrum of textile industrial scraps

Table S3 vibrational bands of FTIR spectrum of textile industrial scraps

wavenumber (cm^{-1})	vibration	compound ID (*)
3334	OH stretch	
1629	COO^- asymmetrical. stretch	Terephthalic acid, disodium salt
1558	COO^- asymmetrical. stretch	
1505	Benzene ring stretch	
1381	COO^- symmetrical. stretch	

1335	Benzene ring stretch
1097	CH in plane bend
1022	C-C stretch
894	CH out-of-plane bend
824	CH out-of-plane bend
742	COO^- in plane bend

* Spectrochimica Acta Part A, 68:817-822 (doi.org/10.1016/j.saa.2006.12.065); NIST Chemistry WebBook, SRD69 - Terephthalic acid, disodium salt

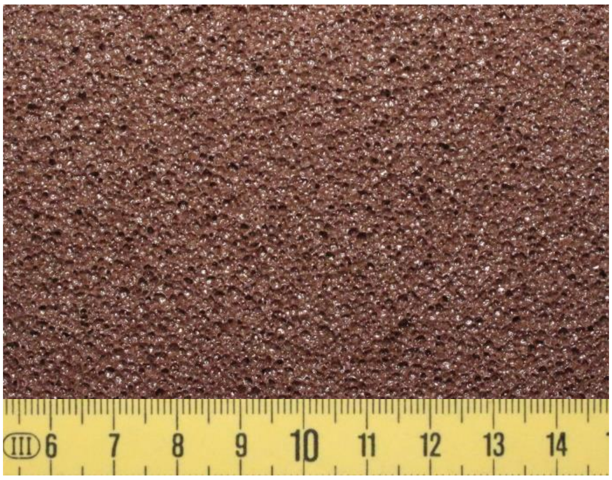

800_30

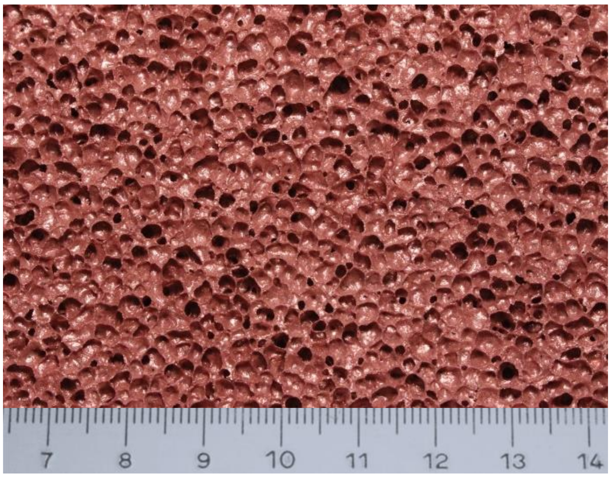

850_30

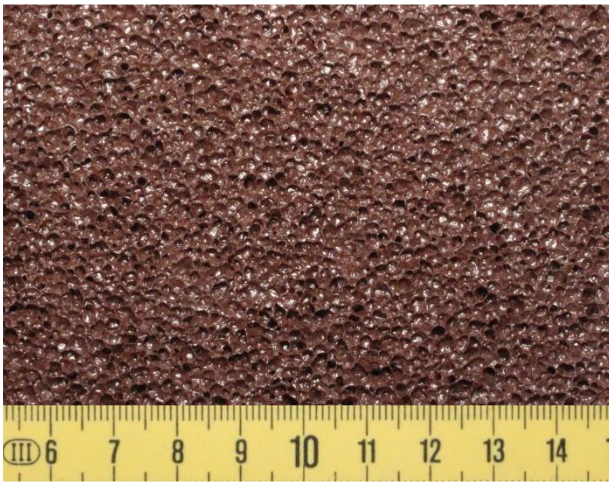

800_45

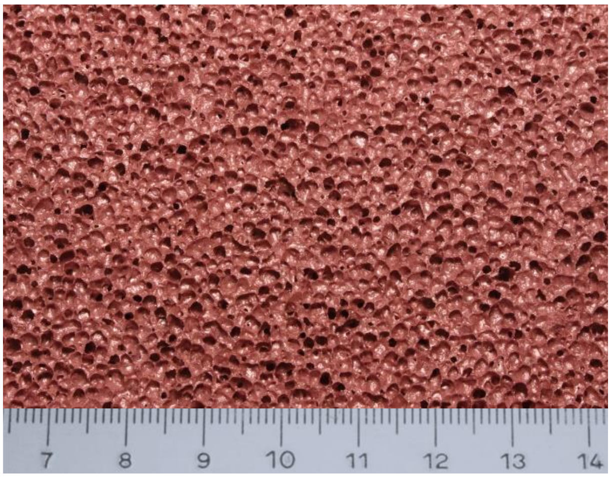

850_45

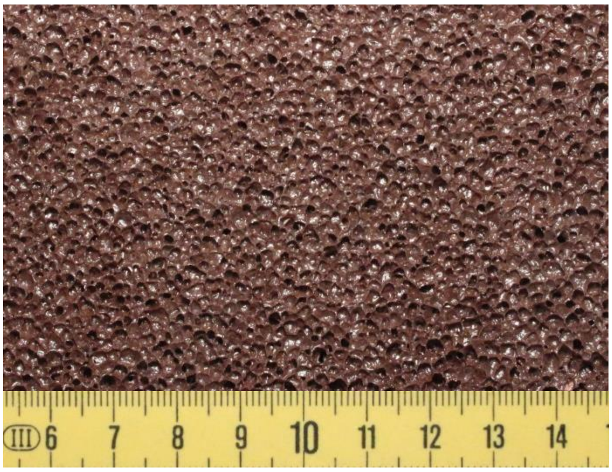

800_90

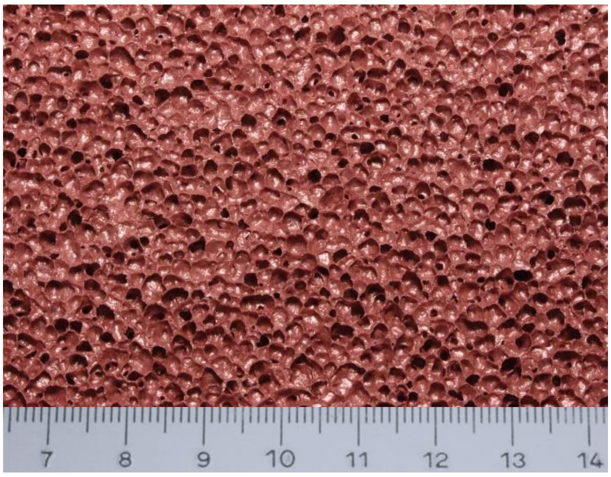

850_90

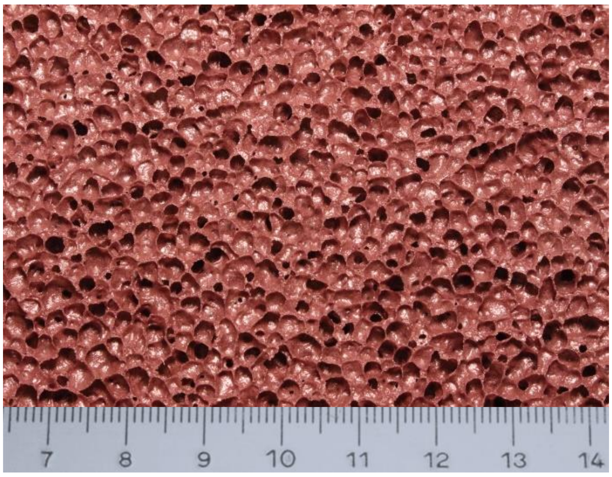

875_30

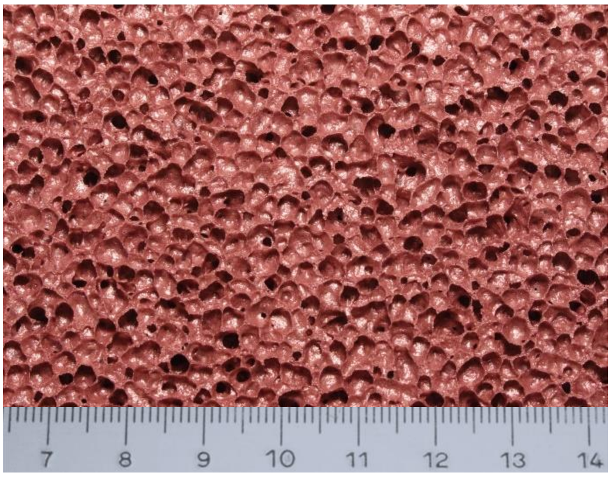

900_30

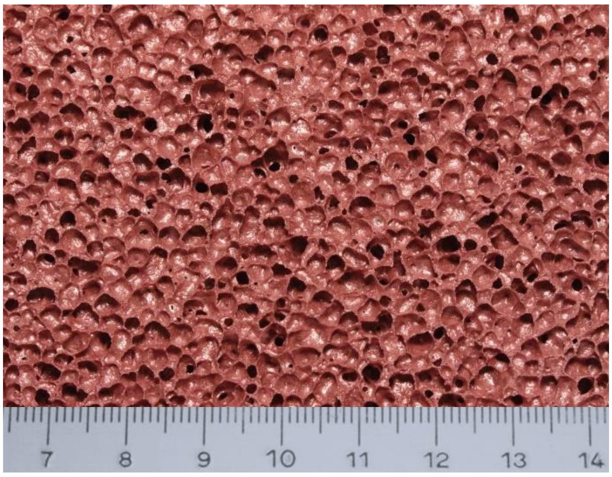

875_45

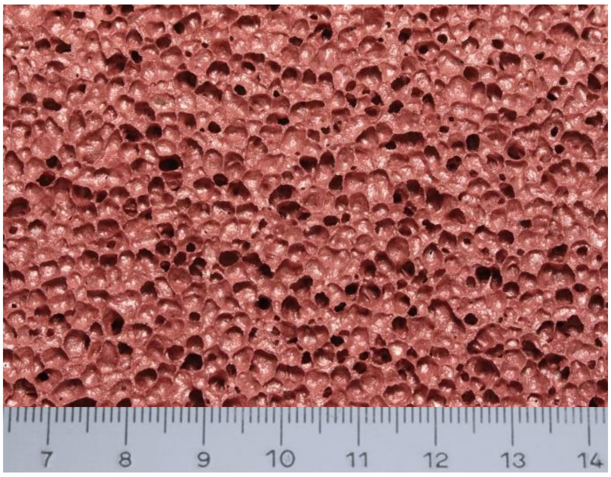

900_45

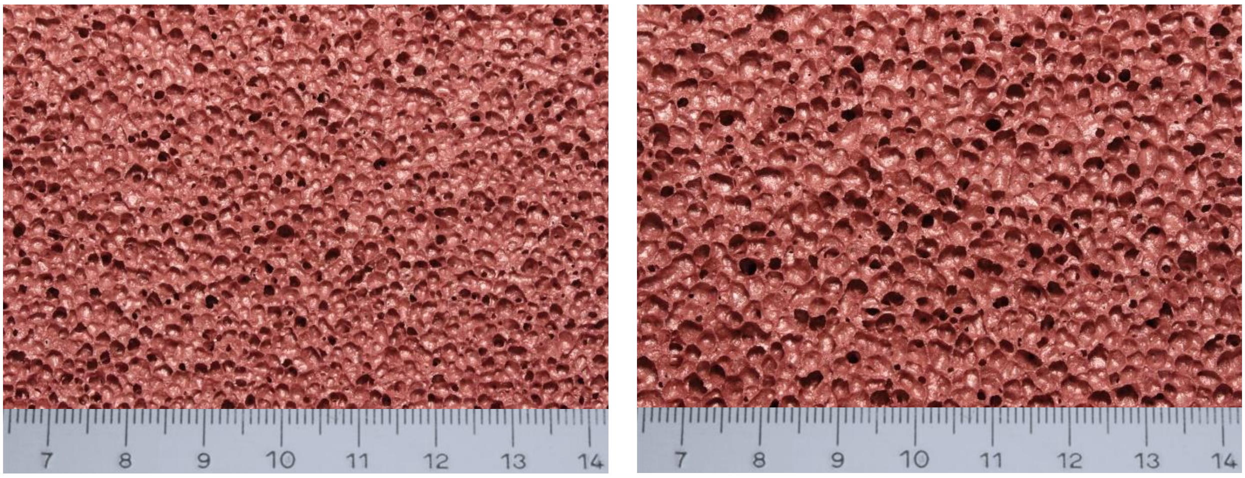

875_90 **900_90**

Figure S3 macrographs of the different samples (sample name below the picture – first number: sintering temperature in °C; second number: sintering time in minutes)

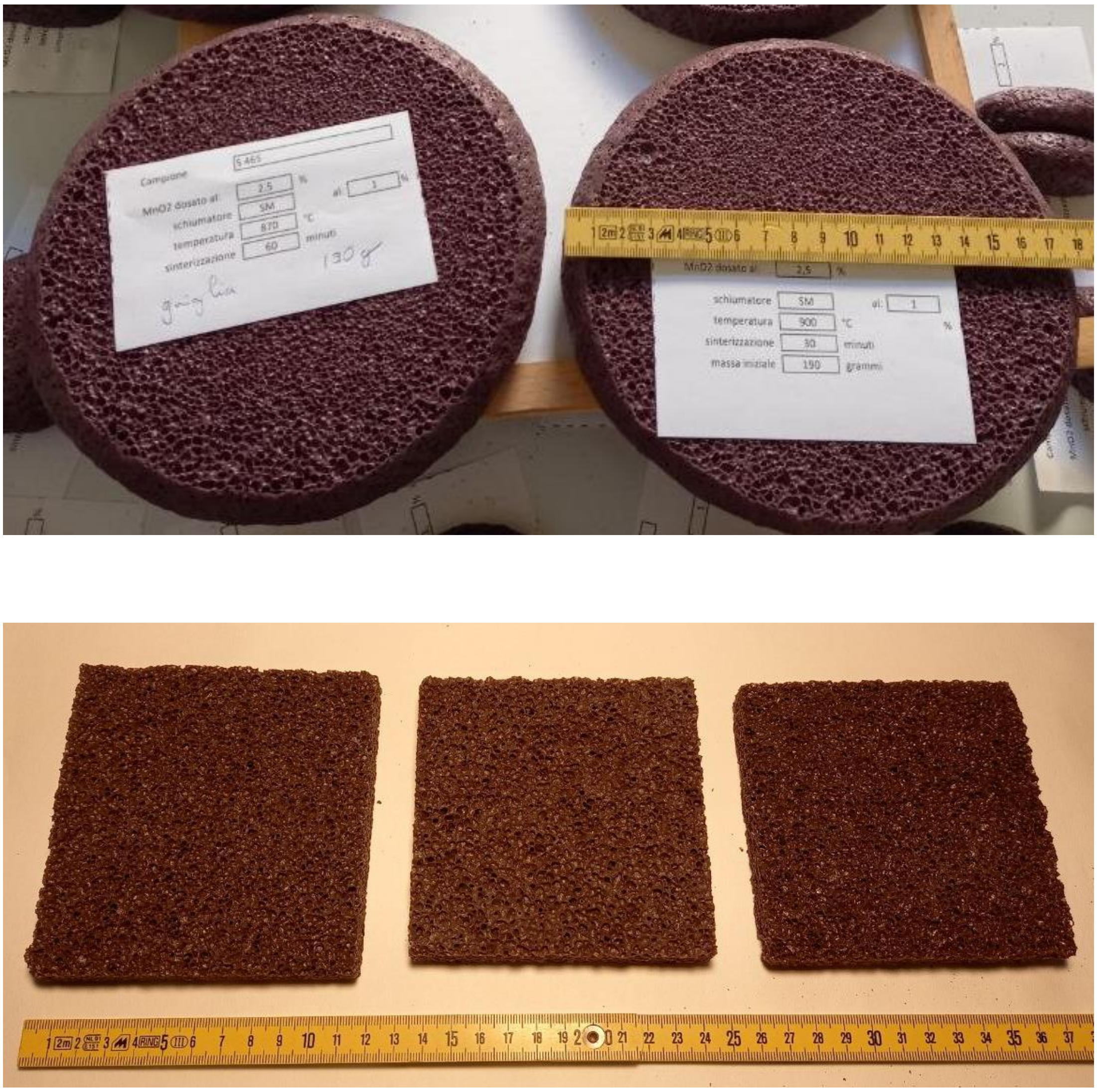

Figure S4 representative pictures of some of produced samples, before and after cutting into final size

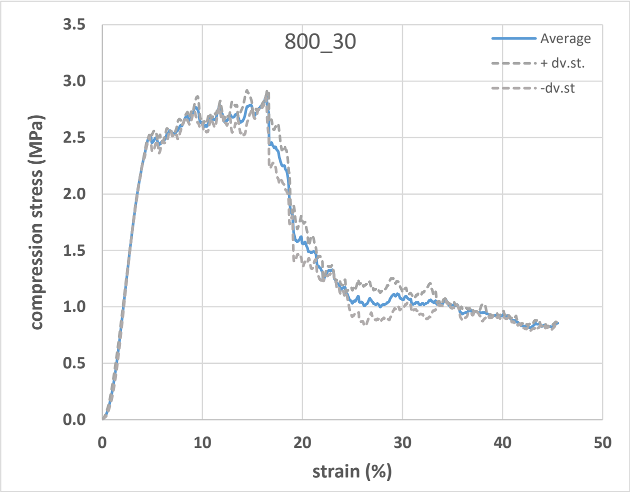

800_30

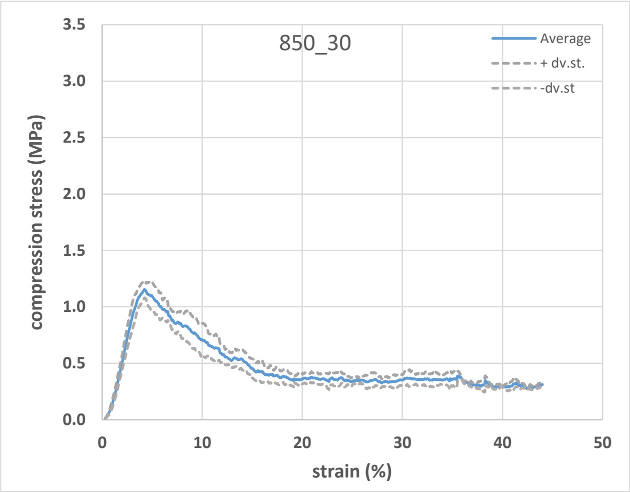

850_30

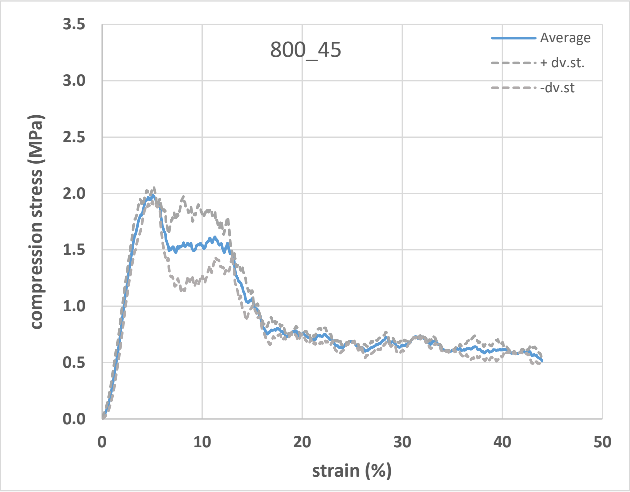

800_45

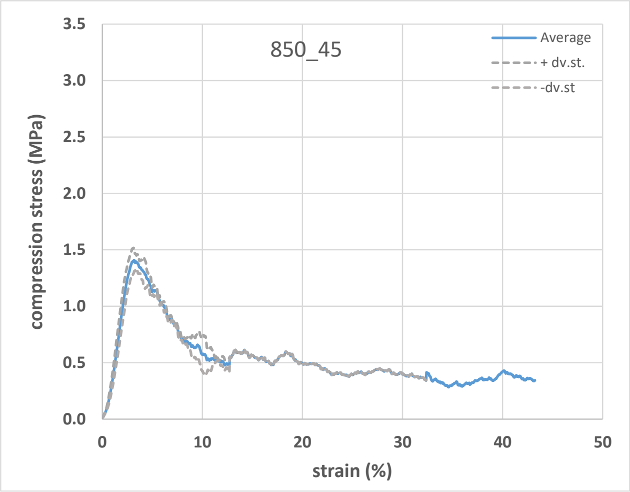

850_45

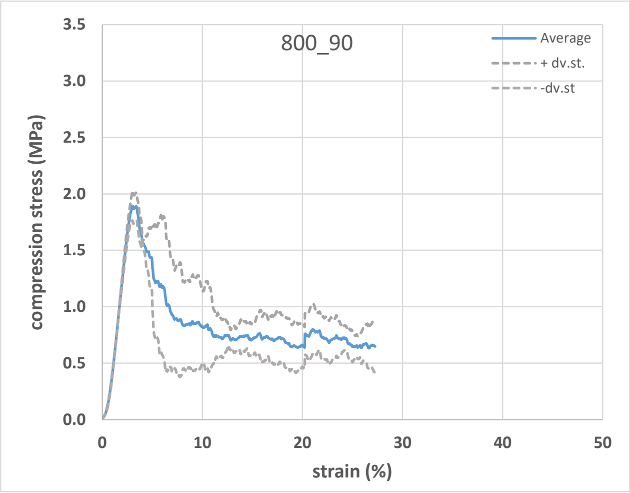

800_90

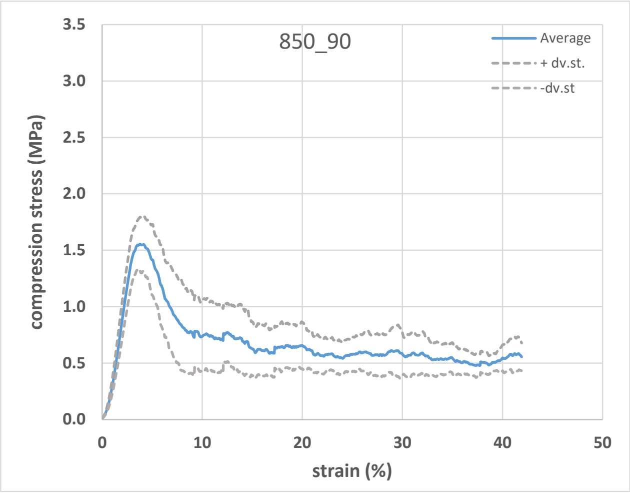

850_90

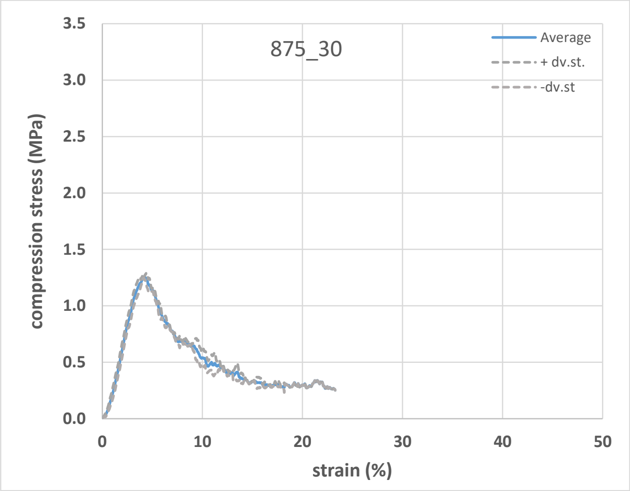

875_30

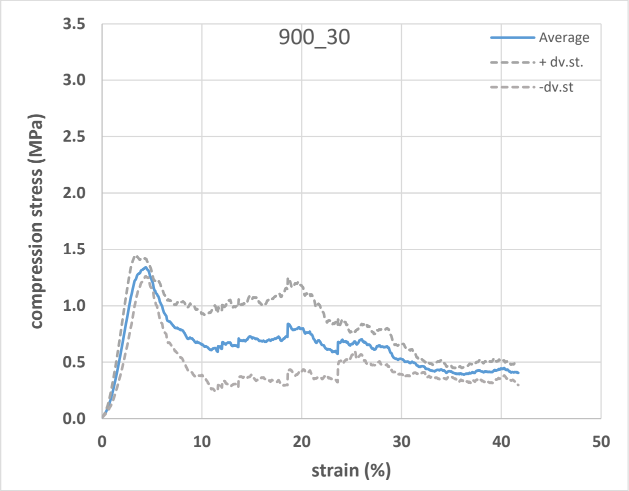

900_30

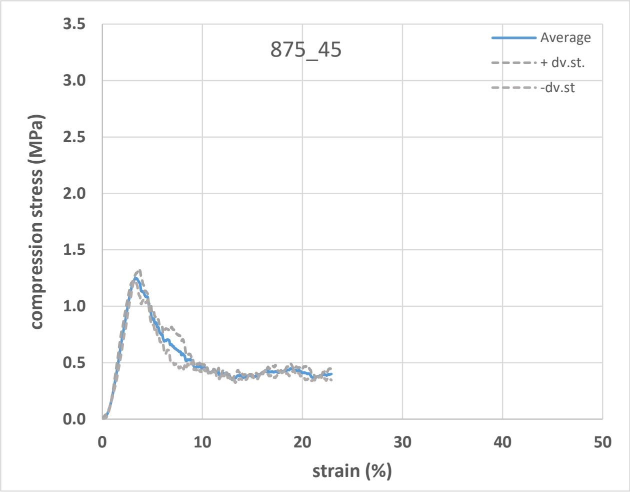

875_45

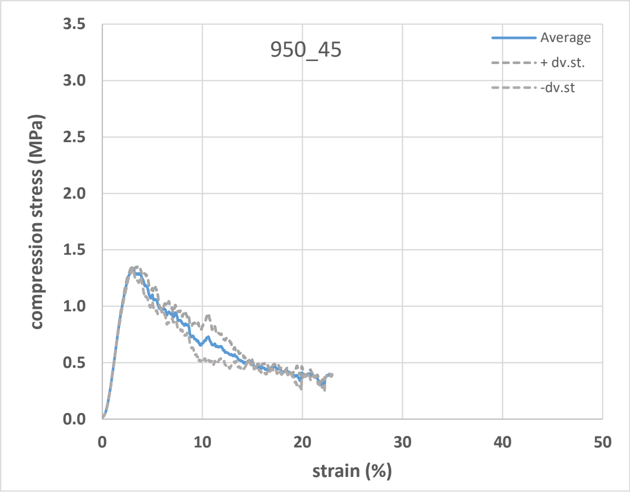

950_45

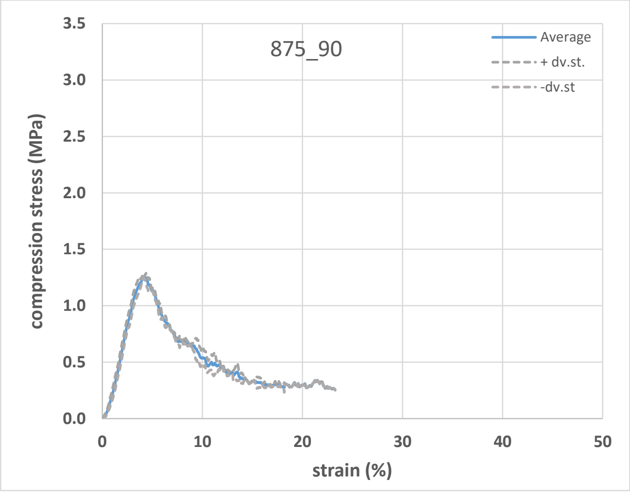

875_90

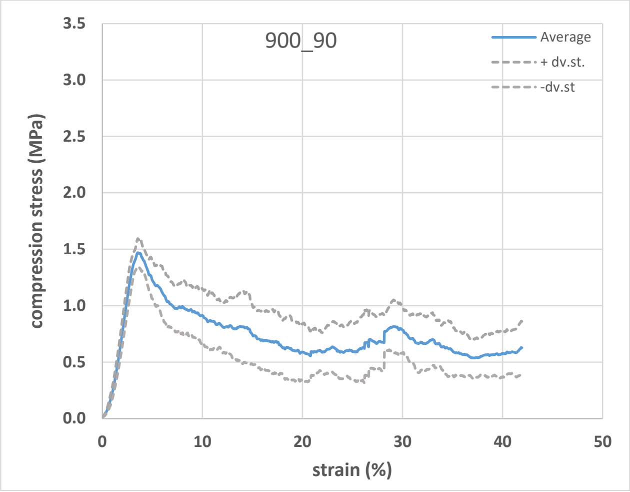

900_90

Figure S5 compression stress/strain curves of different samples (sample name below the picture – first number: sintering temperature in °C; second number: sintering time in minutes)
